# Development of Psychological First Aid Guidelines for People Who Have Experienced Disasters

**DOI:** 10.3390/ijerph182010752

**Published:** 2021-10-13

**Authors:** Eun-Young Kim, Seung-Woo Han

**Affiliations:** 1 Department of Counseling Psychology, Kyungil University, Gyeongsan 38428, Korea; saedew@gmail.com; 2 Department of Emergency Medical Technology, Kyungil University, Gyeongsan 38428, Korea

**Keywords:** development, guidelines, disasters, psychological, first aid

## Abstract

This study developed guidelines for psychological first aid. This guideline promotes core response and disaster capabilities for disaster mental-health professionals, such as mental-health nurses and counseling psychologists at disaster sites. A research team composed of a first-aid professor and counseling psychology professor developed this psychological first-aid guideline to promote the psychological response required at disaster sites. The team verified each question’s content adequacy at each guideline-development stage to determine the appropriateness of response to a disaster. The PFA performance stage and achievement objectives were moved to the next stage only when the research team fully agreed upon them. This guideline revised and supplemented the six steps suggested in the handbook to five steps through expert meetings. The modified part was made into one step, without separating the first rapport formation and safety check. The checklist for evaluation was developed after verification by a total of four people, including one emergency-rescue-department professor, one counseling psychology professor, one paramedic, and one health educator. Based on previous studies, the cutting point is 24 points. The final completed psychological first aid consists of five stages: rapport formation and safety verification, psychological stabilization, information collection, problem resolution, and recovery, with details to be carried out at each step. These guidelines contribute to the promotion of disaster-response capabilities of disaster psychologists. Continuous training and practical exercises based on the five stages will provide fundamental data for a disaster-simulation psychological-first-aid educational development.

## 1. Introduction

Many people worldwide have lost precious possessions (such as homes, health, and property) through natural or human disasters, and in some cases, even their lives have been threatened [1]. As such, catastrophes affect the psychological and physical parts of individuals [2]. Moreover, with the recent increase in earthquakes, fires, and infectious diseases in Korea, the need for emotional care is increasingly urgent [2], because exposure to one-time or repeated traumas can lead to emotional insensitivity or even post-traumatic stress disorder [3]. Therefore, it is essential to understand how to respond in the early stages of a disaster.

According to the Korea Nursing Dictionary, full-scale treatments at specialized institutions implement first aid in advance of the early stages of a disaster. Still, it can significantly impact the recovery of victims [4]. In particular, psychological first aid (PFA) is a short-term care process provided to professional disaster experiences that aims to help stabilize the mind and body, reduce stress and pain, and promote a return to daily life [5]. The sooner the crisis intervention of psychological first aid through counseling is carried out, the sooner the after-effects can be minimized and coping ability can be improved [6].

Furthermore, the Ministry of Public Administration and Security (2020) expanded and implemented “psychological stability and social response standards” to support disaster victims’ emotional reactions and enable them to contribute to the welfare of their community [7]. As a result, disaster psychologists, such as mental-health nurses and counseling psychologists, who care for those exposed to traumatic disaster experiences, are not overwhelmed by extreme stress and require “proper planning and preparation” to cope effectively without panic [8]. In Australia, a three-tiered management framework of treatment and support has been developed by local experts to guide the training and practices of lay personnel and practitioners to plan and meet the diverse mental health needs of people affected by disasters [9]. The United States has continuously attempted to spread the importance of disaster mental health to local communities and maintain the continuity of disaster education by developing an online training module that can improve disaster mental health for disaster personnel [10]. However, there are few standardized disaster mental-health-support training programs dedicated to disaster mental-healthcare workers despite the high number of disasters. This lack makes it difficult to provide efficient services focused on disaster victim psychological support [11].

Therefore, based on research results that people who have experienced disasters experience shock stages within 48 h [6], this study developed PFA guidelines to improve the initial crisis intervention capabilities of mental-health nurses and counseling psychologists for disaster victims. Recently, Korea developed a training program for disaster mental-health support personnel for various direct translation groups that help provide PFA through seminars and hands-on training [2]. However, as the program is limited and not implemented for all relevant institutions and direct translation groups, it is urgent to create a unified PFA guideline focusing on mental-health nurses and counseling psychologists in Korea. Previous studies emphasize the importance of PFA for disaster-response personnel, but providers have diverse jobs and individual levels are inconsistent. Therefore, this research emphasizes that experts on similar levels of PFA capabilities apply to survivors of a consistent method [12]. In previous study [13], people who experience disasters have reactions such as confusion, fear, grief, and loss of confidence. PFA experts should contribute to alleviating their painful emotions and promoting hope and healing. The ultimate goal of the PFA was emphasized to provide the subjects with five principles (promote safety, calm, connection, self-efficacy, and help). In other words, it can mean providing a safe environment to the subject, helping them, improving their self-esteem, and returning to their pre-traumatic level by connecting and utilizing community resources on their own.

Thus, this PFA guideline provides step-by-step implementation goals to implement PFA for those experienced in various disaster situations. This psychological first-aid guideline focuses on psychological recovery from those who have experienced disasters based on post-traumatic growth theory [14]. Post-traumatic growth is a positive psychological change experienced after a trauma event. In this guideline, the step-by-step performance stage was constructed by adding problem-solving, social support, and recovery implied in the post-traumatic growth theory.

## 2. Materials and Methods

### 2.1. Guideline Development Goals

This guideline promotes disaster psychologists’ core response and disaster capabilities, such as mental-health nurses and counseling psychologists at disaster sites. However, disaster psychologists’ knowledge of PFA before is required to apply these guidelines to the subjects. 

PFA prior knowledge include identifying psychological shock reactions, moving the target to mental and physical safe places, exploring disaster events, qualifications related to the expertise of disaster mental-health professionals through listening and empathy, and applying effective communication. The goal of developing the guidelines for first-time emergency services is to help disaster mental-health professionals identify rapport formation and safety, psychologically stabilize targets, and collect incident information from disaster-stricken targets, and improve recovery. 

### 2.2. PFA Guidelines Procedure

The theme of this study is “development of a PFA guideline for people who have experienced natural and social disasters,” developed by a research team which composed of a professor of first aid and a professor of counseling psychology. The research team completed the step-by-step performance step based on the theoretical framework. The research team proposed a draft guideline to overcome the ambiguity of the existing PFA and expand the use of PFA in the community to disaster experts. In addition, this research team is experts that studied post-traumatic growth of occupations that experienced various trauma events. This research team completed the performance stage of each step by expanding the concept of social support and recovery required by post-traumatic growth for the psychological first aid of disaster survivors.

The PFA performance stage and achievement objectives were moved to the next stage only when the research team fully agreed upon. The stage in which a complete agreement was not reached was carried out until the consensus was reached through several meetings and telephone connections, and the next stage was carried out if the two researchers agreed 100%.

This guideline aims for disaster mental-health professionals to implement PFA quickly and consistently. The PFA guidelines are for those who have experienced natural disasters, such as earthquakes, floods, and heavy snow; or social disasters, such as fires, traffic accidents, and building collapse. The guidelines are also for those specialized in mental nursing and counseling psychology at mental-health centers of counseling to license. The PFA guidelines developed in this study mentioned securing a place where survivors can feel psychological stability separated from the risk of disaster sites. If possible, it is important to move survivors of places without irritation such as odors and sounds that reminded them of the scene.

**Figure 1:**
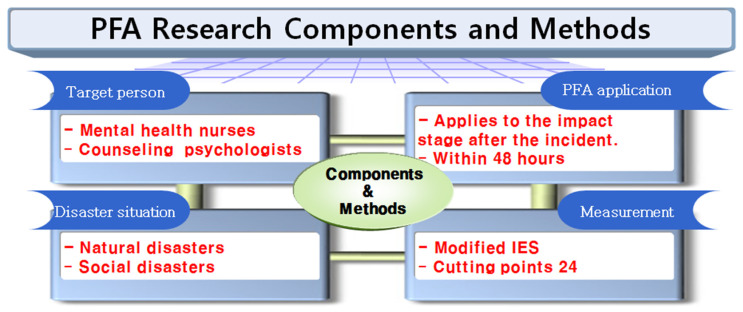
PFA research components and methods.

These guidelines consist of a performance stage, PFA performance objectives at each step, and activities performance objectives at each location as the final principle. These guidelines shall apply PFA within 48 h corresponding to the shock stage after a disaster based on a prior study [2] that an acute intervention within three to seven days from the disaster is essential. The scale used in these guidelines is the additional supplementation of the Impact of Event Scale (IES) originally developed by Horowitz et al. [15], modified by Weiss and Marmar [16] and translated into Korean by Eun et al. [17]. This scale is the most widely used worldwide to date as a self-reporting measure of trauma-related symptoms [18]. Based on Eun et al. [17], we calculated the screening cut-point as 24 (Figure 1, Table 1).

The PFA provider will only perform the procedure if the survivor has given consent to use the checklist, and if the survivor is extremely anxious or nervous, take a short break before proceeding. The PFA provider checks the checklist for any missing items and, if so, encourages survivors to complete it again. PFA providers can actually read the checklist for the elderly who have difficulty reading text or have limited mobility. This scale was used for various cognitive behavioral group therapy. In a study of subjects that experienced post-traumatic stress disorder due to multiple collisions, subjects who received cognitive behavioral group therapy showed low IES after the program [19]. In addition, in a study that applied an art-therapy program to adults who experienced psychological trauma, the IES was significantly lower in the subjects who applied the program. Therefore, it can be confirmed that this scale is an important evaluation tool for verifying the effectiveness of various program interventions [20].

### 2.3. Checklist of Validation and Evaluation of Guidelines

**Figure 2:**
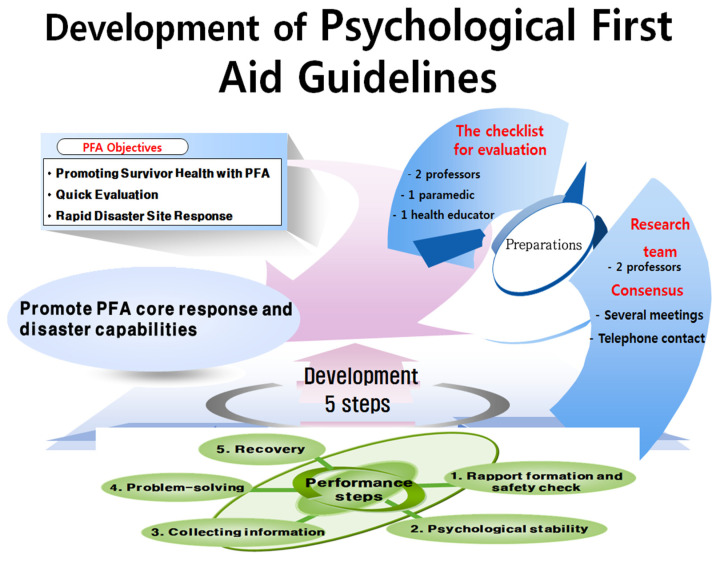
PFA guidelines development process.

This guideline was developed after verification by a total of 4 people including 1 emergency rescue department professor, 1 counseling psychology professor, 1 paramedic, and 1 health educator. In addition, this guideline set the final step by referring to the Handbook for Disaster Nursing and Emergency Preparation [21] and SAMHSA [22]. This guideline is based on the Five Empirically Supported Early Intervention Principles and Eight Core Actions provided by SAMHSA. This guideline unifies practical assistance, connection with social supports, and linkage with collaborative services among the 8 core activities of SAMHSA into problem-solving, and adds a new recovery step to present the final 5 concise PFA guidelines [22]. SAMHSA emphasized the importance of recovery and said that the skills for psychological recovery are in overcoming stress and adversity and reducing pain after a disaster. Therefore, in this guideline, the importance of recovery is emphasized by presenting a PFA achievement goal for each stage, such as “Think of yourself recovering” in the recovery phase. Finally, this guideline checked the psychological shock responses presented in the Handbook for Disaster Nursing and Emergency Preparation [21]; we checked the content adequacy of each step. Next, researchers met with experts to see if the guidelines were appropriate for each step. At each stage, experts agreed on the assessment of discrepancies for items requiring correction and supplementation and secured reliability to achieve 100% agreement (Figure 2 and Table 2). Finally, we applied the evaluation checklist to the actual application of the guidelines in this study.

## 3. Implementation

The guidelines developed in this study solved the ambiguity of PFA core activities suggested in previous study [12]. In other words, the core activities of PFA were set step by step so that disaster experts could easily follow and check them. It is important for disaster mental-health professionals to follow each step not changing the order of the step. In addition, the ambiguity of the previous use of PFA was resolved by clearly classifying disaster experts (mental-health nurses and counseling psychologists) who can apply PFA. The PFA guidelines developed in this study consist of five stages: identification of first rapport formation and safety, psychological stabilization, information collection, problem resolution, and recovery.

The first stage is to verify safety from the physical and psychological standpoint of the target and incident stimuli for the first face-to-face meeting. This stage includes forming a reliable relationship between the disaster psychologist and the target and verifies the target’s psychological response and safety. 

The second stage of these guidelines is psychological stabilization and (1) recalling safe places, (2) applying psychological container techniques, and (3) exploring positive statements. The performance of the activity in the second phase is to isolate the subject’s memory of the place or event related to the disaster situation and verify the value exploration for himself/herself. Those who have experienced a disaster need to stop remembering or thinking for a while because their daily life can be difficult when thinking of trauma-related accidents. Container techniques remind survivors of images of boxes. It is an intervention method that seals unwanted thoughts or memories there and allows survivors to withhold trauma cases until they have psychological freedom [5].

The third stage in these guidelines is to collect information about disaster situations experienced by subjects: (1) the nature of disasters and trauma events, (2) whether they are guilty of loss, (3) whether they are concerned about subsequent threats, and (4) whether they use social resources. The activity is for the disaster psychologist to identify signs of problems and negative emotions by identifying information related to the disaster situation of the target. 

The fourth stage of this guideline is problem-solving: (1) checking what is needed right now, (2) establishing a specific action plan, and (3) using a social-support system. The activities in the fourth stage identify the priorities of the issues that need addressing and ways to use and link internal and external resources. 

Finally, the fifth stage of this guideline is for the target to recall what they look like after a disaster as a recovery step. The last step of the activity is to explore a positive future by identifying the supporting resources.

## 4. Discussion

The PFA guidelines developed in this study enhance the core response and disaster capabilities required by disaster mental-health professionals at disaster sites. The guidelines consist of five stages (rapport formation and safety verification, psychological stabilization, information collection, problem resolution, and recovery), and each stage introduces goals and activities. 

In the first stage, disaster psychologists acknowledge their expertise, stabilize their targets, and confirm physical and psychological safety. In a previous study [23], the fact that the majority of PFA providers evaluated contact and participation with survivors as the most useful core behaviors implies that the formation of trust relationships at the first stage is important. The disaster site is approached by a large number of people with unknown status, where survivors may react negatively [5]. It is important for PFA providers to ensure that survivors are physically safe and psychologically supported. This guideline highlights the stability of the disaster site and suggests that therapeutic communication can be applied for confidentiality. The therapist–consultant interaction was critical in therapeutic rapport and simple communication [24]. This relationship is consistent with disaster mental-health professionals forming a trusting relationship with the whistleblower important in their initial response. Another study [25] also said that counselors create a comfortable environment from danger, giving them relief and bringing up their own stories. In previous study [26], giving immediate physical stability reduces pain and anxiety, and removing various risk factors of survivors increases psychological stability. In particular, if various physical reactions appearing in the shock stage are discovered, the problem can be dealt with quickly by seeking immediate medical care. In this study, based on these previous studies, physical and psychological safety was checked in the PFA achievement goal for each stage in the first stage, and it was suggested to perform psychological shock response check and psychological support of survivors in the activity area.

In the second stage, disaster mental-health professionals remind clients of a safe place, seal memories of unwanted events, and confirm their values by exploring positive statements. Stabilization techniques developed crisis response strategies to control self-management positively [27]. In previous study [28], psychological stability was mentioned in the intervention part as an area of core activity. In particular, this guideline provides time to seal unwanted memories and organize thoughts through container techniques. Another study [5] has shown that survivors spend a lot of time reflecting on invasive memories or thoughts related to trauma, so it is important to postpone the memories of trauma for a while if their lifetime is difficult. 

The stabilization technique proposed in this guideline is consistent in that the party can cope with the crisis by maintaining self-control and sealing unwanted memories from the situation. In a previous study [26], psychological stabilization was said to consist in stabilizing and correcting the unstable minds of psychologically overwhelmed survivors. In particular, it was emphasized as an important core activity for psychological stabilization to respect the privacy of survivors and to give them a few minutes to calmly prepare before the interview. In this study, we also focused on the psychological stability of the survivors by sealing the unwanted shock events and continuously reminding them of a safe place. Therefore, the psychological stabilization techniques proposed in these guidelines contribute to preserving the self-control of the party and improving its crisis capabilities. 

In the third stage, disaster mental-health professionals attempt exploratory questions related to events, identify the loss or guilt caused by the disaster experience, and verify information about the currently available resources. The most important principle of PFA is that it is necessary to flexibly intervene according to the needs of survivors [5]. Appropriate intervention will take place only after sufficient information is collected on survivors. In particular, the behavior of collecting information at most disaster sites can be subject to many restrictions due to limited time. Therefore, it is very important to prioritize information collection in a limited time, and in this guideline, the main information collection order is presented when collecting information. Collecting information is ultimately about gathering additional information to identify immediate needs and concerns. In particular, survivors experience emotions related to experiences such as the “Death of Loved one”, “Losses”, and “Extreme Feelings of Guilt or Shame”, and the persistence of these emotions may lead to future concerns [26]. Therefore, identifying the immediate emotional situation and identifying the currently available resources can prevent a worse situation in advance. In this guideline, “Loss and guilt” and “Confirmation of available resources” were presented in the PFA achievement goals for each stage based on the information collection factors presented in previous studies, and the confirmation factors were presented in detail in the activities. The collection of information presented in this guideline emphasized identifying real-world problems, such as psychological and physical losses caused by disasters, and whether they are at risk later. The guidelines also identified the collection of positive information, such as whether to use social resources. Therefore, the internal counselors’ positive and negative information collection can provide essential data for problem-solving and contribute to establishing future therapeutic directions. 

In the fourth stage, disaster mental-health professionals establish specific action plans and use social-support systems through connection with community counseling centers. Survivors lose hope in a disaster crisis. Helping survivors with what they actually need not only give hope for the future but also improves self-resilience [5]. In other words, since survivors have difficulty logically solving problems in crisis situations, PFA providers must help them express what survivors need. In the post-traumatic growth theory, which is the basis of this guideline, it is said that if social support is properly exercised in extreme emotional pain situations, negative emotions are reduced and the power to overcome is improved [14]. Most people who have experienced trauma are not likely to seek resources such as mental health services and are limited in establishing specific behaviors [29]. The ultimate goal of problem-solving is to help survivors address their immediate needs and concerns in a practical way. In previous study [26], four basic steps were suggested: “Identify the Most Immediate Needs”, “Clarify the Need”, “Discuss an Action Plan”, and “Act to Address the Need”. Each step helps the survivors to take action on their own by talking to the other survivors and making realistic plans to materialize the problem. In this guideline, step-by-step PFA achievement goals are presented so that the action plan can be specified and the immediate needs can be found. In addition, it was made to present what is immediately solvable and what is not possible in the activities, and concrete behavioral goals were specified to be presented to the survivors.

In the final stage, the individuals think about their recovered selves and design a happy future. When the survivor is somewhat stable and immediately problem-solving, it is important for the PFA provider to recognize what survivors should do for themselves later and what subsequent help is important for psychological recovery [5]. A prior study [30] found that methods such as miracle questions, a technique of solution-focused therapy, are meaningful in that they can present resolvable goals and respect for the intruders. Therefore, the guidelines are significant because they promote self-efficacy [31] in reaching the destination and asking for a positive and happy future in solution-driven treatment. In another study, recovery of trauma victims was emphasized [32]. People who experience trauma question their beliefs in the face of dangerous events, but these questions are either more challenged or undergo changes that make them stronger psychologically. In this guideline, based on these previous studies, the individual’s strengths and support resources are re-established by having them perform step-by-step achievement goals and activities, such as “thinking of yourself recovering” and asking “questions about the happy future ahead”.

The significance of these PFA guidelines includes a consistent direction for disaster mental-health professionals. Unlike the existing methods presented in PFA, this finding established different performance stages and specifically describes performance activities at each stage.

This study’s limitation includes its application of PFA to the same age group as children. In addition, this study only applies to disaster mental-health professionals who can understand and carry out disaster situations and disaster mental-health professionals to some extent and cannot apply to experts from other professional groups. However, since PFA is a professional and unique field, it is meant for experts who have worked in disaster psychology centers. Based on this study, measures should be continuously sought to ensure a more practical and professional application of the PFA guidelines. 

Currently, various organizations invest technical support and funds for PFA, and in New York, more than 125 hospitals and 57 local health institutions conduct PFA training [12]. In another previous study, survivors who received PFA in emergency situations were more effective in decision-making than those who did not [33]. Therefore, the latest development and continuous revision of the PFA guidelines provided to experts will contribute to the mental-health well-being of survivors.

## 5. Conclusions

The guidelines developed in this study are for mental-health nurses and counseling psychologists to implement PFA quickly and consistently. The performance goals and activities for each stage required in this guideline are to help PFA intervene early to alleviate the emotional shock of disaster experienced by people and solve necessary problems immediately in order to enable a quick return to daily life. In addition, it can be confirmed that the evaluation of this guideline is being properly performed by applying IES to disaster experiences. 

Until now, various guidelines have been proposed, as the importance of psychological first aid has been highlighted in Korea, but there has been little development of guidelines focused on practitioners. In addition, the PFA guidelines developed so far have been too ambiguous to be used by experts, because no systematic performance stage was presented, but this guideline solved this problem. Finally, this guideline presents “utilization of social-support systems” and “recovery” step by step, based on the post-traumatic growth theoretical framework. This guideline is valuable in that it emphasizes the internal power of trauma-experienced persons emphasized in theory. 

Thus, this study informs the public of the importance of PFA by developing PFA guidelines and further promoting PFA and disaster capabilities of disaster psychologists. Furthermore, the PFA guidelines contribute to developing programs that can apply PFA in various disaster situations.

## Figures and Tables

**Table 1:** Psychological first-aid assessment checklist.

Number	Contents	Never	Sometimes	Usually	Often	Always
1	Reminders of the situation at that time also bring back emotions.	0	1	2	3	4
2	Have trouble keeping up my sleep.	0	1	2	3	4
3	Other things led me to think about the incident.	0	1	2	3	4
4	Felt sensitive and angry after that situation.	0	1	2	3	4
5	Every time I think about the situation, I tried to avoid it because I was confused.	0	1	2	3	4
6	Even if I try not to think, I remember the situation.	0	1	2	3	4
7	Either the situation did not happen, or it did not feel real.	0	1	2	3	4
8	I stayed away from reminders of the situation.	0	1	2	3	4
9	A video of the situation used to pop into my mind.	0	1	2	3	4
10	My nerves became sensitive and I was easily surprised.	0	1	2	3	4
11	I tried not to think about the situation.	0	1	2	3	4
12	I was still confused by the situation, but I endured it.	0	1	2	3	4
13	My feelings for the situation were numb.	0	1	2	3	4
14	There are times when I feel or act as if I were back in the situation.	0	1	2	3	4
15	It was hard to fall asleep after that situation.	0	1	2	3	4
16	I felt a flood of strong feelings about the situation.	0	1	2	3	4
17	I tried to erase the situation from my memory.	0	1	2	3	4
18	I had difficulty concentrating.	0	1	2	3	4
19	Considering the situation, I reacted physically, such as sweating, breathing problems, dizziness, or heart palpitations.	0	1	2	3	4
20	I had a dream about the situation.	0	1	2	3	4
21	I felt I was on guard and observed my surroundings.	0	1	2	3	4
22	I tried not to talk about the situation.	0	1	2	3	4

**Table 2:** Psychological first-aid guidelines: a conceptual framework.

Performance Step	Step-By-Step PFA Achievement Objectives	What to Do with the Activity
1. First rapport formation and safety check	Psychologist’s introduction and confidentiality	Recognize that they are an authorized expert, respecting confidentiality.
	Physical and psychological safety checks	- Check psychological shock reactions (pulse aberration, dizziness, blank eyes, pale complexion, non-controlling urine, etc.) - Ensure feeling of psychological support
	Check safety from incident stimuli	- Go to places that do not have an incentive to recall the scene of the accident.
2. Psychological stability	Recalling a safe place	- Keep my agent in control of themselves.
		- Imagine a safe place to see a relaxed effect.
	Psychological container techniques	- To seal unwanted events by reflecting on trauma-related memories.
	Exploring positive statements	- Explore values for yourself
3. Collecting information	The nature and severity of disasters and traumatic events.	- Ask exploratory questions related to an event - (Where were you at the time of the incident, how afraid were you?)
	Loss and guilt	- Confirmation of depression, anxiety and lethargy due to loss of assets from disaster and trauma - Confirmation of death of family members and acquaintances around them - Check for signs of negative emotions such as extreme guilt - Identification of suicide and murder risk
	Concerns about subsequent threats immediately after the incident	- Check the excess of concern about future risks
	Availability of social resources	- Verify information about the currently available resources
4. Problem-solving	See what you need right now	- Recognize immediate resolution and non-resolution
	Develop specific action plans	- Prioritizing issues to be addressed - Help ensure the realization of agreed goals and specific action plans
	Utilization of social-support systems	- Identification of primary support system (spouse, parents, relatives, friends, etc.) - Link to community counseling centers - Link to additional support services
5. Recovery	Think of yourself recovering	Check the resources I have
		Ask questions about the happy future ahead.

## Data Availability

Data are contained within the article.
